# Application of the PRECEDE -PROCEED model in prevention of brucellosis focused on livestock vaccination process

**DOI:** 10.1186/s12917-021-03099-y

**Published:** 2021-12-13

**Authors:** Farhad Bahadori, Fazlollah Ghofranipour, Fatemeh Zarei, Reza Ziaei, Saeideh Ghaffarifar

**Affiliations:** 1 Department of Health Education and Health Promotion, Faculty of Medical Sciences, Tarbiat Modares University, Tehran, Iran; 2 Department of Health Sciences, Unit for Public Health Sciences, Mid Sweden University, Sundsvall, Sweden; 3 Medical Education Research Center, Health Management and Safety Promotion Research Institute, Tabriz University of Medical Sciences, Tabriz, Iran

**Keywords:** Prevention of brucellosis, PRECEDE-PROCEED model, Educational intervention, Vaccination

## Abstract

**Background:**

This article reports the steps of an educational intervention, which is designed to change livestock breeders’ preventive behavior in terms of vaccinating their livestock against brucellosis. The study has been conducted in a rural area in a country with the second highest brucellosis prevalence in the world.

**Methods:**

In a quasi-experimental study and applying PRECEDE-PROCEED model, 45 livestock breeders were trained through basket method, accompanied with constructive feedback from researchers and peers and a brief interactive lecture at the end. The livestock breeders’ awareness, attitude and practice level in the intervention group was compared with those of other 45 livestock breeders in a control group, 1 and 6 months after the intervention. According to the results of the Rose Bengal tests (RBTs), as a rapid and simple screening test, the presence or absence of Brucella antibodies in the animals’ serum was investigated.

**Results:**

Immediately and 1 month after the intervention, the mean scores of knowledge, awareness and practice of livestock breeders in the intervention group were significantly higher. Six months after the intervention, the results of the RBTs were positive in more livestock in the intervention group compared to the animals in the control group. The positive result of RBT after educational intervention, in livestock whose test results were negative immediately before intervention, accompanying the results of observation indicating a good general condition of livestock was considered as a probable evidence of the success of the educational intervention.

**Conclusion:**

The model-driven educational intervention could significantly increase livestock breeders’ awareness, attitude and practice regarding prevention of brucellosis and vaccination of their livestock against brucellosis; however, a period of non-continuous reinforcement and gradual reduction of the number of the reinforcements by health educator workers is recommended in order to increase the maintenance of the learnt behavior.

**Trial registration:**

Conducting this study was registered at Iranian Registry of Clinical Trials (IRCT20180304038945N1). Registered 24 December 2018. The proposal was registered before enrollment of the first participant.

**Supplementary Information:**

The online version contains supplementary material available at 10.1186/s12917-021-03099-y.

## Background

Brucellosis, as a zoonotic bacterial disease, is a major public health problem in some parts of Asia in the Middle East [1]. The Middle East covers half of the countries with the highest prevalence of Brucella [2]. Brucellosis is one of the important bacterial zoonotic diseases that affects both animals and human beings [3]. While human brucellosis is caused mainly by *Brucella abortus*, the most pathogenic disease in humans is *Brucella melitensis* [3]. This is while *B.melitensis* and *Brucella.suis* are the main causes of brucellosis in cattle, goats/sheep and pigs respectively. Brucellosis can be transmitted to human beings through different ways, including consumption of products of infected animals, direct unprotected contact with body parts or secretions of an infected animal or corps and placentas of aborted livestock [4]. According to the statistics released by WHO, 500.000 people are infected with brucellosis annually [5, 6]. Among this number, 45000 people are residents of eastern Mediterranean, EMRO region [6]. The precise statistics of outbreak, the incidence, and prevalence rate of Malta fever in humans is not available. Because some factors such as various misleading symptoms of the disease among human beings as well as some diagnostic problems affect the precision of diagnostic inferences. Different attempts such as implementation of control and eradication programs help many countries to reduce the number of infections by brucellosis in both humans and animals. However, the prevalence of infection in humans is still high (about 15.4%) in Iran and brucellosis is a serious public concern for Iranian health organizations [6]. Clinical manifestations of human brucellosis in its acute phase (including fever, malaise, anorexia, headache, arthralgia and backache) considerably affect the daily lives of the infected patients. In addition, the complications of the disease, such as arthritis, endocarditis, spondylitis, sacroiliitis, osteomyelitis and meningoencephalitis increase Disability-Adjusted Life Years (DALY) in patients and result in substantial economic losses to livestock breeders [7]. Controlling brucellosis in livestock can be achieved by employment of different methods such as vaccinating livestock, eliminating the infected animals’ products and body parts, and quarantining the animals in the time of buying and selling livestock and transferring them to a community of new livestock in a new stable [7, 8]. Animal vaccination is regarded as a very effective method in reducing the infection among humans [8] and among the above-mentioned methods, livestock vaccination is considered as the first step in brucellosis eradication [9].

Livestock breeders’ insufficient information and awareness about brucellosis transmission pathways, complications of infection, prevention methods and consequences of untreated infection leads to low rate of livestock vaccination, while vaccination is provided free in all provinces and cities of Iran [10]. Emphasizing the importance of livestock breeders’ knowledge and awareness does not mean ignoring the role of other factors, such as inappropriate vaccination time, concerns related to the viability of the vaccine, inappropriate storage conditions of the vaccine, inappropriate quarantine conditions and not predicting of any risk of brucellosis related-abortion among vaccinated livestock [11]. The important point is that the role of most of these factors can be diminished with education. For instance, given that the recommended time to vaccinate livestock against brucellosis is their 3 to 6 months of age, and goats and sheep give birth in winter, livestock vaccination should be done in summer. In this regard, it is necessary to educate livestock breeders that any time other than livestock’s 3 to 6 months of age and other than the summer season is an inappropriate time for vaccination of their livestock against brucellosis.

Considering the insufficient vaccine-related knowledge and awareness of Iranian livestock breeders [12–14], it is recommended to train livestock breeders [15].

To educate livestock breeders, planning for a PRECEDE-PROCEED model-driven vaccination-focused training program can be very useful because theories and models have a significant role in designing, implementation and evaluation of educational programs.

The PRECEDE-PROCEED model as a framework “helps identifying specific intervention targets, allows the integration of individual and environmental factors into one concise program, and includes consideration of organizational, administrative and policy aspects that might hinder or support the practical implementation of a program” [16].

Applying theories or models, educators can analyze the status of a health problem, with participation of the stakeholders and can focus on the most important predictors of their intended behavior and can tailor the education. That is why a PRECEDE-PROCEED model-driven educational intervention can provide an evidence-based framework to intervene [17, 18].

To the best of our knowledge, there has not been published any PRECEDE-PROCEED model -driven intervention focused on livestock vaccination in order to prevent brucellosis so far. Therefore, it was decided to conduct a PRECEDE-PROCEED model -driven intervention focused on livestock vaccination through a training program for the livestock breeders to prevent brucellosis.

This study reports the steps of a quasi-experimental and PRECEDE-PROCEED model-driven interventional study, which is designed to change livestock breeders’ preventive behavior in terms of vaccination of their animals against brucellosis. It was hypothesized that livestock breeders’ awareness, attitude and practice about brucellosis vaccination in the intervention group will be significantly improved and the antibodies will be increased among animals of the livestock breeders in the intervention group.

## Method

This quasi-experimental study was conducted in 2019. It was decided to determine the effects of an educational intervention, based on the PRECEDE-PROCEED model, on changing the awareness, attitude and practice of livestock breeders in prevention of brucellosis and in vaccinating their livestock against brucellosis. The presence of brucellosis antibodies in animals’ blood was examined as the outcome measure of the intervention. Due to the fact that the titer of Brucella antibody increases both after infection and after vaccination, to differentiate between these two conditions, it is recommended to check the type of antibodies secreted (IgM or IgG) in the animal’s serum or perform tests such as 2-mercaptoethanol. Doing polymerase chain reaction (PCR) and even culturing the pathogen as the gold standard method of diagnosis are strongly recommended. However, due to the fact that *Brucella* is a fastidious organism and its culture is not safe and requires a high biosafety level to culture, and due to our financial constraints, in this study, the positive result of Rose Bengal test (RBT) after educational intervention, in livestock whose test results were negative immediately before intervention, accompanying the results of observation indicating a good general condition of livestock was considered as a probable evidence of the success of the educational intervention.

Specific objectives of the study were:

- Comparing the livestock breeders’ awareness level in intervention group with control group, before and after the intervention
- Comparing the livestock breeders’ attitude level in intervention group with control group, before and after the intervention
- Comparing the livestock breeders’ practice level in intervention group with control group, before and after the intervention
- Comparing the presence of anti-brucellosis antibody among livestock in the intervention group with the control group, before and after the intervention

### Setting

The study was conducted in Lighvan, a village, located in the northern slopes of the Sahand Mountain, in the suburb of Tabriz metropolitan city, Iran. In this region, livestock breeders breed mainly sheep and goats and rarely cattle. In addition to animal breeding, they produce most of the country’s dairy products. Animal breeding and dairy production is the main job of Lighvan’s residents. So, Lighvan plays a significant role in cheese production in the country. Lighvan has a population of more than ten thousand people. Each year, one hundred thousand sheep and goats and about 15 thousand lambs and yeanling are bred in Lighvan and at least 2 veterinarians and 2 livestock vaccinators vaccinate the livestock. They observe adherence to health protocols in more than 100 cheese production workplaces in this region.

### Participants

Participants of this study were livestock breeders living in Lighvan. Livestock breeders, who were willing to participate; did not have a history of brucellosis in the last 5 years and had not participated in any similar training courses before, were eligible to be included in this study. Participants with the inability to use the educational package of this study, those who had physical disabilities such as visual or auditory problems, were not included. Being absent in more than 20% of training sessions was an exclusion criterion in this study.

### Sample size calculation

Power & Sample Size Calculator software, version 3.0, was employed to calculate the sample size. The sample size was calculated applying confidence interval of 95%, power of 90%. The findings of a previous study were taken into account too [19]. In this study, it was hypothesized that livestock breeders’ awareness, attitude and practice about brucellosis vaccination in the intervention group will be significantly improved. We planned a study of a continuous response variable from independent control and experimental subjects with 1 control(s) per experimental subject. Expecting the true differences in the experimental and control means of 10, 20 and 25 for livestock breeders’ attitude, awareness and practice, respectively, we should have recruited 31,13 and 5 experimental subjects and 31,13 or 5 control subjects, respectively, in order to confirm the research hypothesis. The final sample size was estimated at 110, considering a 20% drop rate.

### Study population and sampling method

Nearly 1700 livestock breeders work in about 100 dairying centers in Lighvan and 10 to 50 breeders work in every center. Eight centers were included in this study upon their manager’s willingness to participate in this study. In order to decrease the diffusion effect, breeders in the intervention and control groups were selected from different centers. So, those volunteer centers were randomly assigned to intervention or control groups by the research randomizer software. A stratified quota sampling method was employed to determine the exact number of participants from each center [20].

### The steps to design educational intervention

**Fig. 1:**
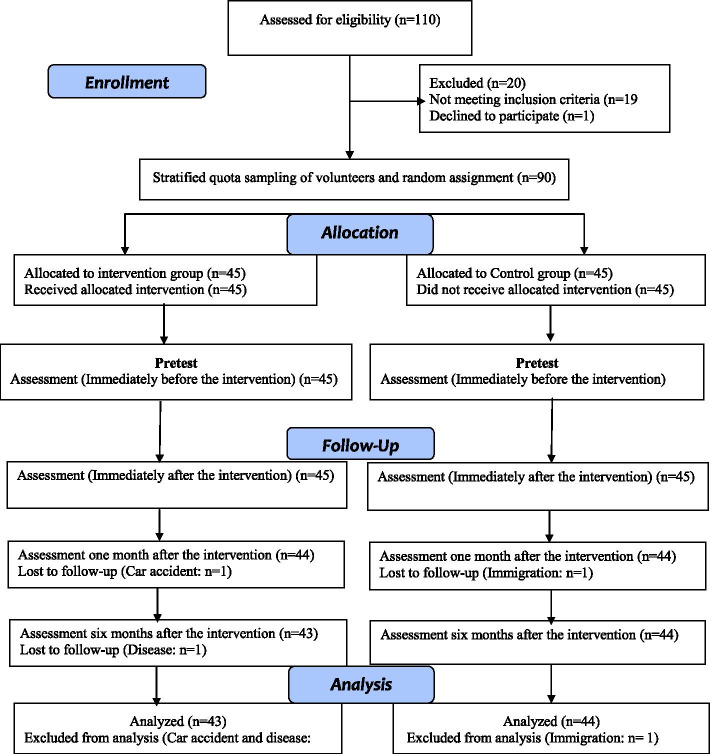
Flow diagram of the study design and enumeration of participants in each step of the study

Applying PRECEDE-PROCEED planning model, an educational package was designed to increase awareness, attitude and practice of livestock breeders in preventing brucellosis by animal vaccination. The steps to design the intervention are summarized in Fig. 1.

To design the educational package, based on the PRECEDE phase of the model, social, epidemiological, educational and ecological assessments were done in Lighvan. By doing so, predisposing, enabling and reinforcing factors of livestock breeders’ vaccination behavior were identified. In this phase, data were collected through semi-structured interviews with all stakeholders, including livestock breeders, veterinarians, vaccinators and health care providers in Lighvan. The data was completed through direct observations of the main researcher (a veterinarian and health education specialist) during 1 month [11].

The BPQ was developed and validated through an exploratory psychometric study. Its initial items were formulated based on main researchers’ observations in the PRECEDE phase of the study, face-to-face interviews (with different stakeholders, including veterinarians, livestock breeders, health educationists and experts from a vaccine and serum production institute in the region) and review of the reports of some worldwide evidence-based best practices. Final version of the BPQ, with 53 items, had acceptable psychometric properties (Content Validity Index = 0.90, Content Validity Ratio = 0.74, Impact Score = 4.30, Intra-class Correlation Coefficient = 0.885, Composite Reliability = 0.895 and standard error of Measurement = 5.448).

Based on the results from exploratory factor analysis, the items of the BPQ were loaded into awareness, attitude, and practice constructs. Awareness items were categorized into three sub-constructs of “direct awareness”, “indirect awareness” and “vaccine oriented awareness”. “The predictive power of awareness, attitude, and practice was determined as 43.43, 15.81, and 15.78% of the livestock breeders’ brucellosis prevention-oriented behavior, respectively” [11].

Additional findings of PRECEDE phase and the process of designing and validating BPQ, as a valid and reliable questionnaire for assessment of the educational intervention in this study, has been published in the first article of this project [11]. BPQ has been attached to this text as Additional file 1.

To design the package for the educational intervention, according to Harden’s six-level program development model, first, learning objectives and then the content of the training package for animal breeders were determined.

The learning objectives and livestock breeders’ educational needs were derived from the assessment results in the PRECEDE phase. The most important predictors of livestock breeders’ vaccination-related prevention behavior were identified based on the results of the factor analysis of the research questionnaire. The most important predictors, which had a greater contribution in determining the educational content, were livestock breeders’ awareness, attitude and practice. Based on the results from exploratory factor analysis, awareness items were categorized into three sub-constructs of “direct awareness”, “indirect awareness” and “vaccine -oriented awareness. The sub-construct with items covering livestock breeders’ awareness about direct transmission pathways of brucellosis, i.e. transmission by livestock’s wool, placenta and fetus, was named as direct awareness. The sub-construct with items covering livestock breeders’ awareness about transmission pathways of brucellosis which occurred without presence of the livestock, i.e. transmission by livestock’s dairy products, was named as indirect awareness. The items of the vaccine-oriented awareness covered livestock breeders’ awareness about all vaccine-related issues.

All items of the BPQ questionnaire are presented in Additional file 1. More training hours were considered to increase livestock breeders’ awareness because the power of livestock breeders’ awareness (43.43%) in prediction of their brucellosis prevention-oriented behavior was higher than the powers of their attitude (15.81%) and practice (15.78%) in predicting the same behavior. The learning objectives and the educational content of the package were discussed and matched with educational needs of participants by the research team. Later, the content was systematically organized into different training sessions. As it is seen in the educational lesson plans, which are presented in the Additional file 2, the training was scheduled in four sessions, including two individual and 40-min face-to-face sessions for each livestock breeder, in the form of a discussion on a selected scenario and photos, with the aim of increasing their knowledge and awareness. The third session was held as a group discussion with the participation of 10–12 livestock breeders in each session and its purpose was to create a positive attitude in livestock breeders to apply the training received in practice. The fourth training session was conducted in a large group with the participation of 40 livestock breeders in the form of a short lecture by the main researcher, questions and answers (Q&A) and summarizing the session by participants. Educational material, including scenarios and pictures for discussion, are presented as the Additional file 3.

The educational content was mainly delivered through the basket method [21]. In this method, some simple and realistic pictures were planned by the research team members. They were taken in the real cultural context of the participants. The findings of a review, that “adding pictures to written and spoken language can increase patient attention, comprehension, recall and adherence” and “patients with very low literacy skills can be helped by spoken directions plus pictures” [21], encouraged us to use the basket method in this study.

Livestock breeders faced many pictures on a board. Pictures included a range of some proper or improper issues about livestock immunization against brucellosis. Livestock breeders were expected to pick up suitable pictures after they heard a scenario. Those scenarios had been written based on the situational assessment data, which was gathered from the PRECEDE phase of the study. Livestock breeders’ choices in selecting the pictures reflected their real-time decisions. Participants received constructive feedback after each individual session. After the individual training was completed in two different sessions, livestock breeders were asked to participate in focus group discussions in order to share their learning and experiences with their peers.

The method for teaching the content was not limited to the basket method and feedback from the researchers and peers. The other training methods and techniques such as Q&A or brainstorming were flexibly employed when they were necessary. The educational intervention was completed through a final mini-lecture at a proper public place and time by the main researcher. Proper educational strategies such as problem-based and integrated learning were adopted to increase the quality of the education as well. Lesson plans for all training sessions and educational scenarios were written and finalized by the research team members.

### The steps to assessment of vaccination rate

The RBT screening test was used to evaluate and compare the vaccination rate of sheep in the two groups. The RBT, as a rapid and simple screening test indicates the presence or absence of *Brucella* antibodies in the animal’s serum. It is interpreted as positive or negative [22, 23]. Due to the fact that the titer of *Brucella* antibody increases both after infection and after vaccination, it is recommended positive sera, particularly those of vaccinated livestock, to be further studied by more specific to comment on the disease status of livestock [22, 23].

For this purpose, in two intervention and control groups, three sheep were sampled from each participating livestock breeder in the study. In the time before vaccination, 135 RBTs were performed in each group. The sheep were randomly selected with software (https://www.randomizer.org). The sampling and the RBT were repeated in 2 to 3 weeks after Brucella vaccination, when the antibody level was at the highest rate. As the research team did not have any authority to follow up the infection status of animals whose antibodies were positive before the intervention and to cull the infected animals, the positive status of the antibodies of those animals were just reported to animal breeders and veterinary officials. Given that up to the time of the second RBT, no action had been taken to eradicate the infected animals, the herd sizes were the same before and after the educational intervention. Vaccination was performed for free by the Veterinary Organization. The positive result of the RBT and the presence of antibodies in the animal’s blood was considered as the success of a livestock breeder in vaccinating his animal [22–24].

### Statistical analyses

Data were summarized and expressed as with frequency and percentage for categorical variables and mean (standard deviation (SD)) for numeric variables. The normal distribution of the numeric variables was assessed by Kolmogorov–Smirnov test. Skewness (within ±1.5 as normal) and kurtosis (within ±2.0 as normal) of the data were assessed too. To compare the baseline variables between intervention and control groups, independent, Mann-Whitney and chi-squared (utilizing an exact procedure) tests were used.

To assess the within group changes over measurements done, the repeated measure analysis of variance (RMANOVA) were used and the group by time interaction effect and the group and time main effects were investigated. The sphericity as an assumption in this analysis was assessed by Mauchly’s test and deviation from the assumption was corrected through Greenhouse-Geiser procedure. To assess the intervention effect i. e. the between group comparisons of changes (immediately after intervention, 1 month after intervention and 6 months after intervention), the analysis of covariance was conducted in two models. In the first model, the baseline measurements were analyzed alone and in the second model, the baseline measures and the other potential confounders were adjusted for variables, including age, occupation, family dimension and education level of the livestock breeders.

The McNemar tests utilizing the exact procedure were utilized to compare the results of the binary outcome i.e. positive/negative RBTs results. The percent of changes were computed too. Fisher’s exact test was conducted to compare the binary outcome at the baseline and logistic regression model was used to model the difference of the binary outcome at after intervention adjusting for baseline measures. Moreover, to assess the effect of the intervention, the absolute risk reduction (ARR) and number needed to harm (NNH) were estimated along with their 95% confidence interval by Newcomb’s method and Bender’s methods for ARR and NNH, respectively [25, 26]. In all analyses, *P* < 0.05 was considered as statistically significant. The analyses were conducted using IBM SPSS Statistics version 25 (IBM corporation, Armonk, USA), STATA 16 (Stata Corp, College Station, Texas, USA) and the graphs were drawn by Graph Pad version 8.3 (www.graphpad.com).

All methods were performed in accordance with the relevant guidelines, regulations and ethical standards of the responsible committee approving the research at Tarbiat Modares University and Iranian Registry of Clinical Trials and with the Declaration of Helsinki, as revised in 2000.

## Results

This quasi-experimental study was conducted with participation of 90 livestock breeders.

Baseline characteristics of study participants are revealed in Table 1. The results indicate that there were no significant differences between intervention and control groups for these characteristics in the baseline (All *P* > 0.05).

**Table 1:** Baseline characteristics of 90 livestock breeders, who participated in a PRECEDE PROCEED model-driven educational intervention

Variables	Intervention (***n*** = 45)	Control (***n*** = 45)	***P***-Value #
**Age** (years) (Mean ± SD)	33.23 ± 10.30	34.13 ± 9.13	0.68
**Animal type(s)** (Frequency (%))			0.50
Sheep & Goat	39 (87.6)	41 (91.1)	
Cow	6 (13.3)	4 (8.9)	
**Job** (Frequency (%))			0.58
Livestock breeder& Another Private job	4 (8.9)	6 (13.3)	
Livestock breeder& Farmer	3 (6.7)	5 (11.1)	
Only livestock breeder	38 (84.4)	34 (75.6)	
**Education** (Frequency (%))			0.41
Illiterate	10 (22.2)	9 (20.00)	
Elementary	20 (44.4)	26 (57.8)	
Not completed high school	12 (26.7)	6 (13.3)	
High school diploma	3 (6.7)	4 (8.9)	
**Number of family members** (Median (P25 – P75))	5 (4–6)	4 (3.5–6)	0.35

#*P*-values are computed based on independent t-, Man-Whitney U and exact Chi-Square tests where appropriate

### Intervention effect

The results of comparing participants’ awareness, attitude and practice scores in intervention and control groups in all time points (before intervention, immediately after, 1 and 6 months after the intervention) are presented in Table 2. Based on the results of covariance analysis in model 1, the effect of intervention was significant on all constructs for all post intervention measurements, 1 and 6 months after intervention (except for “practice” in 6 months after intervention, which the increase was not significant). It seems that the educational intervention has led to an increase in the score of participants’ awareness, attitude and practice in all three measurements taken after intervention (positive mean differences). Furthermore, based on the results of analysis of covariance in model 2, after adjusting for the age, occupation, family dimension and the education level as the potential confounders, the same results were observed for intervention effect except for after 6 months measurement of awareness (Indirect) construct, which the amount of increase was not significant.

**Table 2:** The results of comparing participants’ awareness, attitude and practice scores in intervention and control groups

Variable	Time of measurement	Intervention (***n*** = 43)		Control (***n*** = 44)		MD^**a**^ (95% CI^**b**^)	Model1 ***P***-Value	Model2 ***P***-Value $
		Mean	SD	Mean	SD			
**Direct awareness**	Pre-intervention	23.1	9.2	25.6	9.8	−2.5 (−6.5, 1.5)	0.212 #	–
	Post-intervention	70.4	12.1	26.5	9.6	44.4 (39.8, 48.9)	**< 0.001 ##**	**< 0.001**
	One month after	61.1	16.2	24.8	10.3	36.2 (30.4, 42.0)	**< 0.001 ##**	**< 0.001**
	Six months after	53.5	8.3	24.2	9.3	29.6 (25.9, 33.3)	**< 0.001 ##**	**< 0.001**
**Indirect awareness**	Pre-intervention	40.7	11.4	39.3	14.5	1.4 (−3.9, 6.9)	0.591 #	–
	Post-intervention	68.9	18.4	38.8	15.5	29.6 (22.8, 37.1)	**< 0.001 ##**	**< 0.001**
	One month after	53.6	20.0	39.3	19.1	14.5 (6.3, 22.7)	**0.001 ##**	**0.002**
	Six months after	47.9	18.0	41.0	13.4	7.0 (0.3, 13.7)	**0.040 ##**	0.095
**Vaccine awareness**	Pre-intervention	41.7	13.5	42.8	11.6	−1.1 (−6.4, 4.2)	0.677 #	–
	Post-intervention	67.8	11.0	43.9	11.7	23.8 (19.0, 28.5)	**< 0.001 ##**	**< 0.001**
	One month after	58.5	17.0	44.1	10.9	14.4 (8.4, 20.4)	**< 0.001 ##**	**< 0.001**
	Six months after	54.1	18.6	44.2	12.6	9.6 (3.0, 16.1)	**0.005 ##**	**0.015**
**Awareness in all**	Pre-intervention	35.17	6.68	35.89	8.03	−0.71(−3.8, 2.4)	0.647#	**–**
	Post-intervention	69.01	7.34	36.39	8.30	32.6 (29.3, 35.9)	**< 0.001 ##**	**< 0.001**
	One month after	57.76	8.96	36.07	9.31	21.7 (17.8, 25.6)	**< 0.001 ##**	**< 0.001**
	Six Months after	51.80	9.87	36.48	7.01	15.3 (11.7, 18.9)	**< 0.001 ##**	**< 0.001**
**Attitude**	Pre-intervention	50.9	10.9	48.1	11.9	2.8 (−2.0, 7.6)	0.251 #	–
	Post-intervention	69.7	5.8	49.0	10.1	20.6 (17.1, 27.1)	**< 0.001 ##**	**< 0.001**
	One month after	64.1	9.7	52.2	10.6	11.7 (7.4, 16.0)	**< 0.001 ##**	**< 0.001**
	Six month after	61.2	11.6	50.2	10.4	11.1 (6.4, 15.8)	**< 0.001 ##**	**< 0.001**
**Practice**	Pre-intervention	30.6	6.0	32.4	9.0	−1.8 (−5.0, 1.4)	0.266 #	–
	Post-intervention	59.9	15.1	34.2	10.1	25.6 (20.1, 31.0)	**< 0.001 ##**	**< 0.001**
	One month after	44.3	16.6	33.2	9.0	12.1 (6.6, 17.5)	**< 0.001 ##**	**< 0.001**
	Six months after	36.2	21.5	33.2	7.5	3.8 (−2.8, 10.5)	0.255 **##**	0.338

Bold fonts indicate the significant differences

#*P*-values are computed based on independent t-tests for baseline measurements

##Model 1 *P*-values and MD (95% CI) are computed based on Analysis of Covariance for measurements taken on post, 1 and 6 months after intervention, after adjusting for baseline measurements

$Model 2 *P*-values are computed based on Analysis of Covariance for measurements taken on post, 1 and 6 months after intervention, after adjusting for baseline measurements and potential confounders including the age, occupation, family dimension and the education level

^a^*MD* Mean difference

^b^*CI* Confidence interval

### Time*intervention interaction effect and main time effect

The results of the RMANOVA utilizing the Greenhouse-Geiser correction provided significant interaction effects of time and intervention, which were observed for awareness (direct) (F _(3, 264)_ = 78.03, *P*-Value< 0.001), awareness (indirect) (F _(3, 264)_ = 13.26, *P*-Value< 0.001), awareness (vaccine) (F _(3, 264)_ = 12.20, *P*-Value< 0.001), awareness (total score) (F _(3, 264)_ = 85.015, *P*-Value< 0.001), attitude (F _(3, 264)_ = 12.14, *P*-Value< 0.001), and practice (F _(3, 264)_ = 23.16, *P*-Value< 0.001)). Therefore, the time trends of the measurements were different in the intervention group as compared to the control group. In the intervention group, a raise in the score of each construct is observed immediately after intervention but the amount of difference decreased over time at 1 and 6 months after the intervention. Besides, the time effect was significant for all constructs (All *P*-Value< 0.001).

The results of Sidak post hoc test showed significant pairwise differences among measurements in intervention group for awareness (direct), awareness (indirect), awareness (vaccine), attitude and practice constructs (All *P*-Values< 0.05), but the differences were not significant in control group (All *P*-Values> 0.05) (the means of constructs remained the same over time).

### Between group comparisons of baseline the Rose Bengal tests results

The results of Fisher’s exact test showed that there was no significant difference between scores of presence of antibodies in intervention and control groups at baseline (*P* > 0.05, positive cases about 2.2% versus 1.5% in intervention and control groups).

The results for between and within group comparisons of baseline measurements of the Rose Bengal test are shown in Table 3.

**Table 3:** Between and within group comparisons of baseline measurements of antibody

Presence	Time	Intervention (***n*** = 135 livestock)	Control (***n*** = 135 livestock)	***P***-Value#
**Positive**	Before	3 (2.2%)	2(1.5%)	1.000
	After	93 (68.9%)	62(45.9%)	**< 0.001**
	Change Statistics: McNemar’s Chi2 (1), Exact P-Value	84.38, **< 0.001**	60.0, **< 0.001**	
**Negative**	Before	132 (97.8%)	133(98.5%)	1.000
	After	42 (31.1%)	73 (54.1%)	**< 0.001**
	Change Statistics: McNemar’s Chi2 (1), Exact P-Value	84.38, **< 0.001**	60.0, **< 0.001**	

Data area expressed as n (%)

Significant *P*-Values are shown in bold

#*P*-Value based on Fisher’s exact test

### Within group comparison of the Rose Bengal test results

The results of McNemar’s exact test showed that there were significant changes in scores of in both the intervention and control groups (Both *P* < 0.001, 66.7% changes in the intervention group vs 44.4% in the control group in positive/negative test results). The results of logistic regression to compare intervention and control group’s vaccination after intervention measures of antibody adjusted for before intervention measures are presented in Table 4.

**Table 4:** Results of logistic regression to compare intervention and control group’s vaccination after intervention measures of antibody adjusted for before intervention measures

Group	Odds Ratio	95% CI Lower	95% CI Upper	***P***-Value
Control	Referent			
Intervention	2.632	1.598	4.335	**< 0.001**
**Before Intervention Measure**				
Negative	Referent			
Positive	0.423	0.066	2.703	0.363

Significant *P*-Values are shown in bold

*CI* Confidence Interval

### Between group comparisons of after intervention Rose Bengal tests results

The results of logistic regression after controlling antibody for baseline measures showed that there was significant difference between the intervention and control groups (OR = 2.632, 95% CI: 1.598–4.335, *P*-Value< 0.001).

### Estimating pure intervention effect by NNH

The results of intervention effect assessment revealed an ARR = 0.24 95% CI (0.13–0.35). These results indicate that the intervention controls the adverse events by 24%. NNH = 4, 95% CI (2.83–7.89) indicates the point that in order to prevent brucellosis in one livestock, four livestock breeders should receive training to vaccinate their livestock .

## Discussion

According to the findings of this quasi-experimental study, the PRECEDE-PROCEED model-driven educational intervention led to significant increases in livestock breeders’ awareness, attitude and practice regarding prevention of brucellosis and vaccination of their livestock against brucellosis. Immediately after and 1 month after the intervention, the mean scores of knowledge, awareness and practice of livestock breeders in the intervention group were significantly higher than the mean of these scores in the control group. Six months after the educational intervention, the results of the RBTs were positive in more livestock in the intervention group compared to the animals in the control group. These results confirm that livestock breeders in the intervention group, under the model- driven training, behaved better in preventing brucellosis and vaccinating their livestock.

The role of education in increasing the adoption of appropriate behaviors to prevent brucellosis in livestock and humans [27] or the effect of livestock vaccination in reducing the incidence of brucellosis in humans [28] have been already investigated. In most of the previous studies, educational interventions were either scheduled solely on the basis of researchers’ knowledge and experience, or were planned based on other models of behavior change, such as Health Belief Model (HBM) [8, 29, 30]. As it is explained by Manoj Sharma, HBM is not “culturally versatile [31]. It does not account for cultural factors and socioeconomic status of the target group [31]. Moreover, it “is not about changing health behavior but only explaining it” [32]. In this regard, it is recommended to conduct similar studies in different cultural and socioeconomic contexts to examine the ability of similar PRECEDE-PROCEED-driven interventions to produce other desired context-related results.

Previously, the PRECEDE-PROCEED model had been employed to focus on only preventative behaviors, such as wearing appropriate personal protective equipment, pasteurization of dairy products, consumption of well-cooked meat, sanitary handling and disposal of animal tissues and aborted fetuses [12, 19, 33], not livestock vaccination. To the best of our knowledge and up to the time of writing this article, no PRECEDE-PROCEED-based study has been conducted in the field of brucellosis prevention, which has focused on the combination of health education and vaccination. Taking collaborative and interdisciplinary effective actions, such as “vaccine campaigns, community outreach and education” has been recommended to decrease the burden of brucellosis and its prevalence in endemic regions [34]. This recommendation is in line with the findings of this study that interventions are better to focus on the combination of health education and vaccination, not a single intervention alone.

In our study, the PRECEDE-PROCEED model, as one of the most popular participatory planning models [35] which has been applied to design educational programs in different disciplines [36–38], successfully helped us to provide a tailored road map for designing a participatory intervention program through an approach which started with the desired outcome of the presence of brucellosis antibody in the blood of the livestock of the trained livestock breeders. The significant difference in the presence of antibodies in the blood of livestock in the intervention group compared to the control group confirmed the efficacy of PRECEDE-PROCEED-driven interventions in improving preventive behaviors. As the structure of the PRECEDE-PROCEED model is designed to assess health and quality of life needs [36], it is recommended to examine the impact of such interventions on improving the health and quality of life of participating livestock breeders through additional longitudinal studies in the future.

One and 6 months after the intervention, examining the continuity of the impact of the trainings provided revealed that livestock breeders’ scores in the intervention group were still higher than these scores in the control group; however, the trends of livestock breeders’ scores were declining. Moreover, although 6 months after the intervention, the mean scores of indirect awareness and practice of livestock breeders in the intervention group were higher than the mean of these scores in the control group, those differences were not statistically significant (*P* > 0.05). The declining trend of the scores and the non-significant difference in the scores of livestock breeders in the intervention and control groups 6 months after the intervention reminds the need for continuous training. In order to maximize the external validity of the findings of this research, in terms of maintenance or long-term effects of the educational program [39], it is recommended to incorporate livestock breeders’ behavior into a period of non-continuous reinforcement after ensuring that preventive behavior is learned by livestock breeders. It means that health educators need to gradually reduce the number of the reinforcements until the behavior becomes controlled by stimuli in livestock breeders’ natural environment.

In this study, the educational content was delivered mainly through the basket method. Using pictures in this method helped improve our mostly illiterate livestock breeders’ comprehension because the pictures showed relationships among their ideas and beliefs. This finding is consistent with the findings of a review that “adding pictures to written and spoken language can increase patient attention, comprehension, recall and adherence” and “patients with very low literacy skills can be helped by spoken directions plus pictures” [40]. As it is highlighted in this review, sensitivity to the culture of the participating livestock breeders in creating the pictures has been one of the success factors in our intervention. This alignment in paying attention to the culture of participants as a success factor of intervention can be attributed to using the participatory model of the PRECEDE-PROCEED in designing the intended educational intervention.

## Conclusions

In this PRECEDE-PROCEED model-driven study, the educational intervention led to significant increases in livestock breeders’ awareness, attitude and practice regarding prevention of brucellosis and vaccination of their livestock against brucellosis. According to the findings, the basket method was effective in delivering the educational content for the illiterate participating livestock breeders. A period of non-continuous reinforcement and gradual reduction of the number of the reinforcements by health educator workers until the livestock breeders’ behavior become controlled by stimuli in the natural environment is recommended in order to increase the maintenance of the learnt behavior. In this regard, the cooperation of the pertinent public and governmental institutions and many interdisciplinary actions are needed to train and persuade livestock breeders that vaccinating their livestock is crucial in preventing brucellosis in both livestock and human beings.

### Limitations of the study

There were some limitations in the process of design, implementation and evaluation of the educational intervention in the current study since all livestock breeders living in the study setting were men. Most of the participants in this study were illiterate or had received a very low level of education. Hence, the main researcher had to explain all questions to the participants and complete the research questionnaires by himself. All details about questions were explained such that livestock breeders could understand it. To ensure that the illiterate livestock breeders fully understand the meaning of each question, the researcher had to explain further about each of the questions. In addition he had to check his inference about their answer to each question in order to be sure that he had recorded exactly the answer they intended in the answer sheet. Doing all these steps required more time for explanation for each participant. In this regard, repetition of the study with participation of literate and female livestock breeders may have different results.

Moreover, this study was conducted with participation of the volunteer diary production centers (workplaces). Although it was tried to randomly allocate the volunteer diary production centers to the intervention and control groups, it was not possible to randomly assign the livestock breeders of each center to different groups. Because the livestock breeders under the auspices of each center were meeting each other on a daily basis and by assigning them to two different groups, it was possible to exchange information between the livestock breeders of the control and intervention groups. It was not possible to recruit participants from two different villages. There were only two villages with similar context to research in the study region and we had to develop and validate the research questionnaire (BPQ), with participation of animal breeders at one of them, which is different from the one considered for the educational intervention. In future studies, if the participants of the intervention and control groups are selected from two probable different villages and participants from each center are randomly assigned to different groups; more valid results can be achieved.

In this study, it was decided to evaluate the effect of the educational intervention based on an objective criterion. In this regard, compared to the Polymerase Chain Reaction (PCR), which was very specific, while expensive and not affordable for the research team, the presence of antibodies based on the RBT was considered sufficient in this study. Indeed, measuring the presence of antibodies in the blood of vaccinated animals with the RBT (without determining antibody level) was the only option feasible to the research team in Iran. To evaluate the effect of the similar educational interventions in future studies, the infection and immunization status of brucellosis in cattle’s serum can be complimented by more specific tests such as checking the type of antibodies secreted (IgM or IgG) in the animal’s serum or performing tests such as 2-mercaptoethanol or PCR. PCR is one of the routine detection tests for some fastidious bacteria such as *Brucella* [41]. “While PCR directly detects the DNA of the pathogen, serology is dependent upon the rising and falling titers of antibodies during the different phases of brucellosis” [42]. “Animals that are positive for DNA only may be in the incubation period before an antibody titer develops or may simply be unable to produce specific antibodies at all” [42].

### Strengths of the study

The educational intervention in this study is the first PRECED-PROCEED-driven intervention with an evidence-based emphasis on brucellosis prevention based on animal vaccination. The intervention has been designed and implemented with the participation of all stakeholders (livestock breeders, health educationists, veterinarians and experts from vaccine and serum production institute). The Educational content and the assessment questionnaire were tailored based on needs and situational assessment. Method triangulation (literature review, the main researcher’s field notes and face-to- face interviews with different stakeholders) was employed to do needs and situational assessments.

## Supplementary Information

**Additional file 1.** Brucellosis Prevention Questionnaire (BPQ).

**Additional file 2.** Educational lesson plans.

**Additional file 3.** Educational material, including scenarios and pictures for discussion.

## Data Availability

All data and materials will be available on reasonable request from the corresponding author.

## Abbreviations

DALY: Disability Adjusted Life Year
FAO: Food and Agriculture Organization
HBM: Health Belief Model
IRCT: Iranian Registry of Clinical Trials
MTM: Multi-Theory Model
NNH: Number Needed to Harm
PRECEDE: Predisposing, Reinforcing and Enabling Constructs in Educational Diagnosis and Evaluation
PROCEED: Policy, Regulatory, and Organizational Constructs in Educational and Environmental Development
Q&A: Question and Answer
RBT: Rose Bengal Test
SPSS: Statistical Package for Social Sciences
WHO: World Health Organization

## References

[1] Harisson’s, Circulatory AIN, Functions R, Vascular P (2015). Princi P Les of I Nternal Medicine.

[2] Nematollahi S, Ayubi E, Karami M, Khazaei S, Shojaeian M, Zamani R (2017). Epidemiological characteristics of human brucellosis in Hamadan province during 2009–2015: results from the National Notifiable Diseases Surveillance System. Int J Infect Dis.

[3] Mirnejad R, Jazi FM, Mostafaei S, Sedighi M (2017). Epidemiology of brucellosis in Iran: a comprehensive systematic review and meta-analysis study. Microb Pathog.

[4] Hasanjani Roushan MR, Ebrahimpour S (2015). Human brucellosis: an overview. Casp J Intern Med.

[5] Geng L, Feng Y, Li D, Nan N, Ma K, Tang X (2020). Meningoencephalitis, coronary artery and keratitis as an onset of brucellosis: a case report. BMC Infect Dis.

[6] Bagheri H, Tapak L, Karami M, Amiri B, Cherghi Z (2019). Epidemiological features of human brucellosis in Iran (2011-2018) and prediction of brucellosis with data-mining models. J Res Health Sci.

[7] Musallam II, Abo-Shehada MN, Hegazy YM, Holt HR, Guitian FJ, Hegazy YM (2016). Systematic review of brucellosis in the Middle East: disease frequency in ruminants and humans and risk factors for human infection. Epidemiol Infect.

[8] Alimohammadi M, Bidarpour F, Sharafi H, Ghasemi SM, Zahraei A, Karimyan K (2016). Design and determine the validity and the reliability of brucellosis education questionnaire based on health belief model. Int J Pharm Technol.

[9] Hashemi Tabar G, Jafari A (2014). Preventive and control programs for brucellosis in human and animals. J Zoonoses.

[10] Esmaeili H, Sciences KA-RJ of M, 2013 U. The effects of brucellosis vaccination in domestic animal on human brucellosis in IRAN. rjms.iums.ac.ir. http://rjms.iums.ac.ir/article-1-2662-en.html. Accessed 24 Mar 2019.

[11] Ghofranipour F, Bahadori F, Ghaffarifar S, Ziaei R (2020). Design and validation of brucellosis prevention questionnaire focused on animal vaccination. BMC Public Health.

[12] Hajari A, Shams M, Afrooghi S, Fadaei Nobari R, Abaspoor Najafabadi R (2016). Using the precede-proceed model in needs assessment for the prevention of brucellosis in rural areas of Isfahan, Iran. Armaghane-danesh, Yasuj Univ Med Sci J.

[13] Mahmoodabad SSM, Barkhordari A, Nabizadeh M, Ayatollahi J (2008). The effect of health education on knowledge, attitude and practice (KAP) of high school students’ towards brucellosis in Yazd. PLoS One.

[14] Shahrbabaki PM, Asadi A, Imani Z, Khanjani N (2014). The effect of training on students regarding the prevention of Brucellosis. Rep Health Care.

[15] Kansiime C, Atuyambe LM, Asiimwe BB, Mugisha A, Mugisha S, Guma V (2015). Community perceptions on integrating animal vaccination and health education by veterinary and public health workers in the prevention of brucellosis among pastoral communities of south western Uganda. PLoS One.

[16] Bammann K, Recke C, Albrecht BM, Stalling I, Doerwald F (2021). Promoting physical activity among older adults using community-based participatory research with an adapted PRECEDE-PROCEED model approach: the AEQUIPA/OUTDOOR ACTIVE project. Am J Health Promot.

[17] Ghaffarifar S, Ghofranipour F, Ahmadi F, Khoshbaten M (2015). Why educators should apply theories and models of health education and health promotion to teach communication skills to nursing and medical students. Nurs Midwifery Stud.

[18] Glanz K, Rimer BK (2005). Theory at a glance: a guide for health promotion practice.

[19] Oruogi MA, Bayt Asghari A, Charkazi A, Jvaheri J (2012). Survey on effect of health education intervention on reduction of brucellosis incidence in rural areas of Khomein based on PRECED framework. J Health.

[20] Acharya AS, Prakash A, Saxena P, Nigam A, Acharya AS (2013). Sampling: why and how of it? Symposium sampling: why and how of it?. Indian J Med Spec.

[21] Yakovleva NO, Yakovlev EV (2014). Interactive teaching methods in contemporary higher education. Pacific Sci Rev.

[22] Ducrotoy MJ, Bardosh KL (2017). How do you get the Rose Bengal Test at the point-of-care to diagnose brucellosis in Africa? The importance of a systems approach. Acta Trop.

[23] Esmaeili H, Partovi R, Marhamati Khamemeh B, Hamedi M, Khaji L (2012). Evaluation of the national sheep and goat brucellosis control program in Iran. J Arak Univ Med Sci.

[24] Farazi AA, Hosseini SD (2012). Diagnostic validity of the conventional brucellosis serological tests in. Arak Med Univ J.

[25] Bender R (2001). Calculating confidence intervals for the number needed to treat. Control Clin Trials.

[26] Newcombe RG (1998). Interval estimation for the difference between independent proportions: comparison of eleven methods. Stat Med.

[27] Khanian HR, Hashemian AH (2013). Effect of training on preventive behavior of brucellosis. Iran J Health Educ Health Promot.

[28] Jelastopulu E, Bikas C, Petropoulos C, Leotsinidis M (2008). Incidence of human brucellosis in a rural area in Western Greece after the implementation of a vaccination programme against animal brucellosis. BMC Public Health.

[29] Shahnavaz M, Esmaili F, Masoudi G, Ansari-Moghadam A, Raeisy D, Khashei F (2016). Preventive behaviors of brucellosis in Khash city ranchers based on health belief model in 2015. Iran J Health Educ Promot.

[30] Karimy M, Montazeri A, Araban M (2012). The effect of an educational program based on health belief model on the empowerment of rural women in prevention of brucellosis. Arak Med Univ J.

[31] Sharma M (2015). Multi-theory model (MTM) for health behavior change. WebmedCentral Behav.

[32] Eskandari Z, Bashirian S, Barati M, Soltanian AR, Hazavehi SMM (2017). The effect of educational program based on the health belief model on brucellosis preventive behaviors among traditional ranchers in rural areas of Hamadan province. J Educ Community Health.

[33] Jahangiry L, Khazaee-Pool M, Babazadeh T, Sarbakhsh P, Ponnet K (2020). A community-based randomized trial for the prevention and control of brucellosis among rural populations in Iran: application of the PRECEDE planning model.

[34] Franc KA, Krecek RC, Häsler BN, Arenas-Gamboa AM (2018). Brucellosis remains a neglected disease in the developing world: a call for interdisciplinary action. BMC Public Health.

[35] Glanz K, Rimer BK, Viswanath K (2008). Health behaviour and health education.

[36] Crosby R, Noar SM (2011). What is a planning model? An introduction to PRECEDE-PROCEED. J Public Health Dent.

[37] Binkley CJ, Johnson KW (2013). Application of the PRECEDE-PROCEED planning model in designing an oral health strategy. J Theory Pract Dent Public Health.

[38] Garcia ML, Gatdula N, Bonilla E, Frank GC, Bird M, Rascón MS (2019). Engaging intergenerational Hispanics/Latinos to examine factors influencing childhood obesity using the PRECEDE-PROCEED model. Matern Child Health J.

[39] Dzewaltowski DA, Estabrooks PA, Glasgow RE (2004). The future of physical activity behavior change research: what is needed to improve translation of research into health promotion practice?. Exerc Sport Sci Rev.

[40] Houts PS, Doak CC, Doak LG, Loscalzo MJ (2006). The role of pictures in improving health communication: a review of research on attention, comprehension, recall, and adherence. Patient Educ Couns.

[41] Mobed A, Baradaran B, de la Guardia M, Agazadeh M, Hasanzadeh M, Rezaee MA (2019). Advances in detection of fastidious bacteria: from microscopic observation to molecular biosensors. TrAC Trends Anal Chem.

[42] Gwida MM, El-Gohary AH, Melzer F, Tomaso H, Rösler U, Wernery U (2011). Comparison of diagnostic tests for the detection of Brucella spp. in camel sera. BMC Res Notes.

