# Supplementary material for: Application of the PRECEDE -PROCEED model in prevention of brucellosis focused on livestock vaccination process
Source: BMC Vet Res. 2021 Dec 13;17:384. doi: 10.1186/s12917-021-03099-y (PMC8667392; doi:10.1186/s12917-021-03099-y)
Supplement: Supplementary file 2 — Additional file 2. Educational lesson plans. [file 12917_2021_3099_MOESM2_ESM.docx]

| Goal: to increase the knowledge and awareness of the livestock breeders about transmission pathways of brucellosis | | | | | |
| --- | --- | --- | --- | --- | --- |
| **Session number: One** | **Subject of the meeting: Prevention of brucellosis** | **Target group: Livestock breeders**  **Number: One(face-to-face meeting)** | **Educational context: Cheese production center** | **Training duration:**  **40 minutes** | **Instructor:**  **Farhad Bahadori**  **veterinarian** |

| **Learning objectives:**  **At the end of this session, the learner will be able to:** | **Learning domain/ level** | **Educational content delivery method** | **Assessment method** |
| --- | --- | --- | --- |
| Name the pathways of disease transmission to the humans | Cognitive / Understanding | Face to face training, Providing explanations on the subject based on the scenarios, Basket method, Providing constructive feedback to the livestock breeders by the researcher | Researcher’s observational assessment, Completing the checklist, livestock breeder’s reflection and feedback, Livestock breeders’ photo choices |
| List examples of livestock diseases causes. | Cognitive / Understanding |  |  |
| Choose the images related to the disease transmission pathways | Cognitive / Application |  |  |
| Express their current attitude towards prevention of brucellosis. | Cognitive / Evaluation |  |  |

**Lesson plan of the first educational session**

**Researcher evaluation checklist**

| **Researchers evaluation checklist** | **yes** | **no** | **Total score** |
| --- | --- | --- | --- |
| Pays attention and discusses with the researcher during the training session |  |  |  |
| Expresses his weaknesses in discussion with the researcher |  |  |  |
| Points out his strengths in discussion with the researcher |  |  |  |
| Criticizes his belief around superstitions about transmission of the disease to humans |  |  |  |
| Chooses the correct photos |  |  |  |

**Lesson plan of the second educational session**

| Goal: to change the beliefs and awareness of livestock breeders about prevention and direct transmission pathways of brucellosis | | | | | |
| --- | --- | --- | --- | --- | --- |
| **Session number: Two** | **Subject of the meeting: Prevention of brucellosis** | **Target group: Livestock breeders**  **Number: One(face-to-face meeting)** | **Educational context: Cheese production center** | **Training duration:**  **40 minutes** | **Instructor:**  **Farhad Bahadori (**  **Veterinarian, PhD student)** |

| **Learning objectives:**  **At the end of this session, the learner will be able to:** | **Learning domain/ level** | **Educational content delivery method** | **Assessment method** |
| --- | --- | --- | --- |
| 1- Explain the importance of vaccination as one of the prevention ways. | Cognitive / Remembering | Face to face training, Providing explanations on the subject based on the scenarios, Basket method, Providing constructive feedback to the livestock breeders by the researcher | Researcher’s observational assessment, Completing the checklist, livestock breeder’s reflection and feedback, Livestock breeders’ photo choices |
| Criticize his wrong beliefs and view. | Cognitive / Understanding |  |  |
| Recognize the pictures related to the ways of preventing the disease and explain about them. | Cognitive / Application |  |  |

**Researcher evaluation checklist**

| **Researchers evaluation checklist** | **yes** | **no** | **Total score** |
| --- | --- | --- | --- |
| Pays attention and discusses with the researcher during the training session |  |  |  |
| Expresses his weaknesses in discussion with the researcher |  |  |  |
| Points out his strengths in discussion with the researcher |  |  |  |
| Criticizes his belief around superstitions about transmission of the disease to humans |  |  |  |
| Chooses the correct photos |  |  |  |

**Lesson plan of the third educational session**

| **Goal: to create a positive attitude in livestock breeders (predisposing structures) in order to feel responsible for disease prevention and vaccination as the first step of prevention** | | | | | |
| --- | --- | --- | --- | --- | --- |
| **Session: three** | **Sessions topic: Prevention of brucellosis** | **Target group: livestock breeders**  **Number: 10-12 people** | **Educational context: Cheese production center** | **Training duration:**  **60 Minutes** | **Farhad Bahadori (**  **Veterinarian, PhD student)** |

| **Learning objectives:**  **At the end of this session, the learner will be able to:** | **Learning domain/ level** | **Educational content delivery method** | **Assessment method** |
| --- | --- | --- | --- |
| Share their experiences with peers about disease prevention. | Affective / Responding | Focus group discussion with brainstorming in the  session, Q&A | Assessment of verbal and non-verbal communication of livestock breeders while negotiating with others |
| Criticize each other's views on the importance of the disease prevention and livestock vaccination. | Affective / Valuing |  |  |
| Argue the necessity of prevention and vaccination in proposed scenarios. | Affective/ Valuing |  |  |
| Explain their responsibility towards the brucellosis prevention | affective/ Organization |  |  |

| **Researchers evaluation checklist** | **yes** | **no** | **Total score** |
| --- | --- | --- | --- |
| Share their experiences with peers about disease prevention. |  |  |  |
| Criticize each other's views on the importance of the disease prevention and livestock vaccination. |  |  |  |
| Argue the necessity of prevention and vaccination in proposed scenarios. |  |  |  |
| Explain their responsibility towards the brucellosis prevention |  |  |  |

**Lesson plan of the fourth educational session**

| Goal: to improve the performance of livestock breeders in disease prevention and livestock vaccination | | | | | |
| --- | --- | --- | --- | --- | --- |
| **Session: four** | **Sessions topic: Prevention of brucellosis** | **Target group: livestock breeders**  **40 people** | **Educational context: Cheese production center** | **Training duration:**  **60 Minutes** | **Farhad Bahadori (**  **Veterinarian, PhD student)** |

| **Learning objectives:**  **At the end of this session, the learner will be able to:** | **Learning domain/ level** | **Educational content delivery method** | **Assessment method** |
| --- | --- | --- | --- |
| Compare their previous and current performance related to brucellosis prevention. | Psychomotor/ guided response | Large group discussion with brainstorming in the session, Mini-lecture | Analysis of verbal and non-verbal communication of livestock breeders while negotiating with others |
| Compare their performance with the ideal requested one | Psychomotor/guided response |  |  |
| Make suggestions to improve their performance regarding prevention of the disease | Psychomotor/Mechanism |  |  |
| Offer suggestions to strengthen the cooperation and coordination among livestock breeders, vaccinators and veterinarians. | Psychomotor/Mechanism |  |  |

| **Information sources for education** | **The necessary equipment for education** |
| --- | --- |
| 1. http://brc.umsha.ac.ir/uploads/brucellosis_national_guide.pdf 2. <https://shepmodel.tbzmed.ac.ir/Page/43/SHEP-model-%DA%86%DB%8C%D8%B3%D8%AA%D8%9F.html> 3. <file:///C:/Users/sagha/Downloads/396604.pdf> 4. Real educational photos prepared by the researcher in the research field | A cardboard for installing the photo |
