# Supplementary material for: Application of the PRECEDE -PROCEED model in prevention of brucellosis focused on livestock vaccination process
Source: BMC Vet Res. 2021 Dec 13;17:384. doi: 10.1186/s12917-021-03099-y (PMC8667392; doi:10.1186/s12917-021-03099-y)
Supplement: Supplementary file 3 — Additional file 3. Educational material, including scenarios and pictures for discussion. [file 12917_2021_3099_MOESM3_ESM.docx]

**Scenario 1:**

Mr. Ligvani is one of the main livestock breeders in Ligvan village. Last summer, he and his young son Ali went to their neighbor villages' livestock market and bought some seemingly healthy sheep and added them to their herd. Mr. Ligvani was waiting for the sheep to give birth in early winter. Ali loved sheep very much and played with sheep in the barn for a long time. A number of sheep had abortions before the calving season. Mr. Ligvani was very upset about this.

**Question: If you were Mr. Ligvani, what would you do to prevent your animals from having an abortion? Please choose the photos which represent your intended actions. You need to explain the reason for each of your choices.**

**Scenario 2:**

Mr. Ligvani with the help of his son Ali collected the aborted fetuses and their placentas by their hands. They gave them to their guard dog to eat. Ali was used to helping his father in milking and he was used to drinking a glass of fresh milk immediately after milking. After a while, Ali had fever, sweats and chills, headache, joint and back pain. He was taken to the village health center and then was referred to a hospital. After many tests at the hospital, it was confirmed that Ali has gotten brucellosis.

**Question: Select images which are related to Ali's misbehavior and explain why those behaviors were wrong? If you were Mr. Ligvani, how would you teach your child about prevention of brucellosis? Select the photos related to those prevention actions and separate them from the rest.**

**Scenario 3:**

Mr. Ligvani's son has been hospitalized because of brucellosis adverse effects. Today, Ali’s general appearance was indicating the seriousness of his clinical problems. For this reason, Mr Ligvani was very sad. On the way back from the hospital, Mr. Ligvani saw his neighbor. The neighbor told him that the veterinary department would come tomorrow to vaccinate all livestock in the village against brucellosis. Mr. Ligvani said that he may not get his livestock vaccinated this year because of concerns surrounding Ali's recent hospitalization.

**Question: Do you think Mr. Ligvani's reasons for not vaccinating his livestock against brucellosis are correct? Why?**

**Scenario 4**:

Today, it was announced in the village that the veterinary department will come for vaccination of the livestock against brucellosis. Mr. Ligvani has prepared all the sheep, lambs and goats for vaccination. He has a must do thing and he has to leave the village to do that urgent job in the city. He cannot wait for hours to get his livestock vaccinated. He is in doubt whether to leave the vaccination job to his nephew or not. His nephew has just returned from military service and has nothing special to do.

**Question: If you were Mr. Ligvani, what decision would you make? Why?**

**Some of the photos are grouped in a file. They can be accessed following this link:**

<https://anyflip.com/xnsxt/qups>
